# Synovial sarcoma of the tibial nerve - case report of a rare tumor in a rare location requiring early diagnosis

**DOI:** 10.1186/s12883-023-03061-5

**Published:** 2023-02-10

**Authors:** Pavlína Hemerková, Hana Matulová, Martin Vališ, Jiří Soukup, Martin Kanta, Jiří Jandura

**Affiliations:** 1 Department of Neurology, University Hospital Hradec Králové, Hradec Králové, Czech Republic; 2 The Fingerland department of Pathology, University Hospital Hradec Králové, Hradec Králové, Czech Republic; 3 Department of Neurosurgery, University Hospital Hradec Králové, Hradec Králové, Czech Republic; 4 Department of Radiology, University Hospital Hradec Králové, Hradec Králové, Czech Republic

**Keywords:** Synovial sarcoma, Rare tumor, Tibial nerve, Case report

## Abstract

**Background:**

We present the case of a patient with a rare synovial sarcoma (SS) of the tibial nerve. So far, only 4 cases of patients with SS originating from the tibial nerve have been described in the literature, and our patient is only the second patient whose limb was saved during treatment. Synovial sarcomas are malignant mesenchymal tumors, i.e., tumors arising from connective tissue. Synovial sarcomas account for 5–10% of all soft tissue sarcomas. However, the name synovial sarcoma is misleading, because the tumor does not originate from synovial cells, but rather from primitive mesenchymal cells. The name most likely originated from the localization around the large joints on the limbs, more often on the lower ones, in the area of the knee joints.

We point out the aspects of correct and quick diagnosis and subsequent treatment, which has very important effect on the patient’s prognosis. Primary less radical excision without prior biopsy verification leads to a higher risk of local recurrence, even if a proper reexcision was performed immediately after biopsy verification of the sarcoma.

**Case presentation:**

A woman born in 1949 began to suffer at the end of 2020 with escalating pain under the left inner ankle with a projection to the sole and fingers. Her personal, family work and social history were insignificant. After the initial neurological examination, the patient was sent for an ultrasound examination of the ankle, which showed a lobular mass measuring 50 × 22 × 16 mm and according magnetic resonance imaging, the finding appeared to be a suspicious neurinoma of the tibial nerve. The tumor was surgically excised, without prior biopsy verification: a 50 × 20 mm tumor was dissected in the distal part of the tarsal canal, which grew through the structure of the tibial nerve and in some places into the surrounding area and appeared intraoperatively as a neurofibroma. But histologically the tumor was classified as monophasic synovial sarcoma. The patient was indicated for a wide reexcision of the skin with the subcutaneous tissue of size 91 × 20 × 15 mm. Now the patient is being treated with external radiotherapy to the tumor bed and she is able to walk.

**Conclusion:**

This report draws attention to a rare type of malignant nerve tumor, which both clinically and radiologically can mimic benign peripheral nerve sheath tumors. Synovial sarcoma should be considered in very painful resistances, typically located around the joints of the lower limbs, the growth of which can be slow. Because the size of the tumor is a negative prognostic factor, it is necessary to make a timely diagnosis using MR imaging and a biopsy with histological examination and to start treatment quickly. Surgical treatment should take place only after a biopsy with histological examination of the tumor so that it is sufficiently radical and does not have to undergo an additional reoperation, as happened in the case of our patient.

## Backround

We present the case of a patient with a rare synovial sarcoma (SS) of the tibial nerve. So far, only 4 cases of patients with SS originating from the tibial nerve have been described in the literature, and our patient is only the second patient whose limb was saved during treatment [1–4].

## Case presentation

A woman born in 1949 began to suffer at the end of 2020 with escalating pain under the left inner ankle with a projection to the sole and fingers, which therefore corresponded to the sensitive innervation region of the tibial nerve. The pain was severe, she could hardly touch the area around her ankle because of the palpation sensitivity. It worsened when she stepped on it, forcing her to walk on her tiptoes. If we were to evaluate the pain using a numerical scale (Numeric Rating Scale, NRS) with a range of 0– 10, or 0– 100, where “+” means “no perceived pain” and 10 (or 100) represents “worst imaginable pain”, the patient’s reported value was around 8.

So far, the patient has been treated for arterial hypertension, bronchial asthma, and diabetes mellitus type II, gastroduodenal ulcer disease, in 1985 she underwent right nephrectomy for clear cell carcinoma and in 1999 hysterectomy with adnexectomy for myomatous uterus.

The patient’s mother died of an unspecified gynecological malignancy. Otherwise, the family history was negative oncologically. Work and social history were insignificant.

**Fig. 1:**
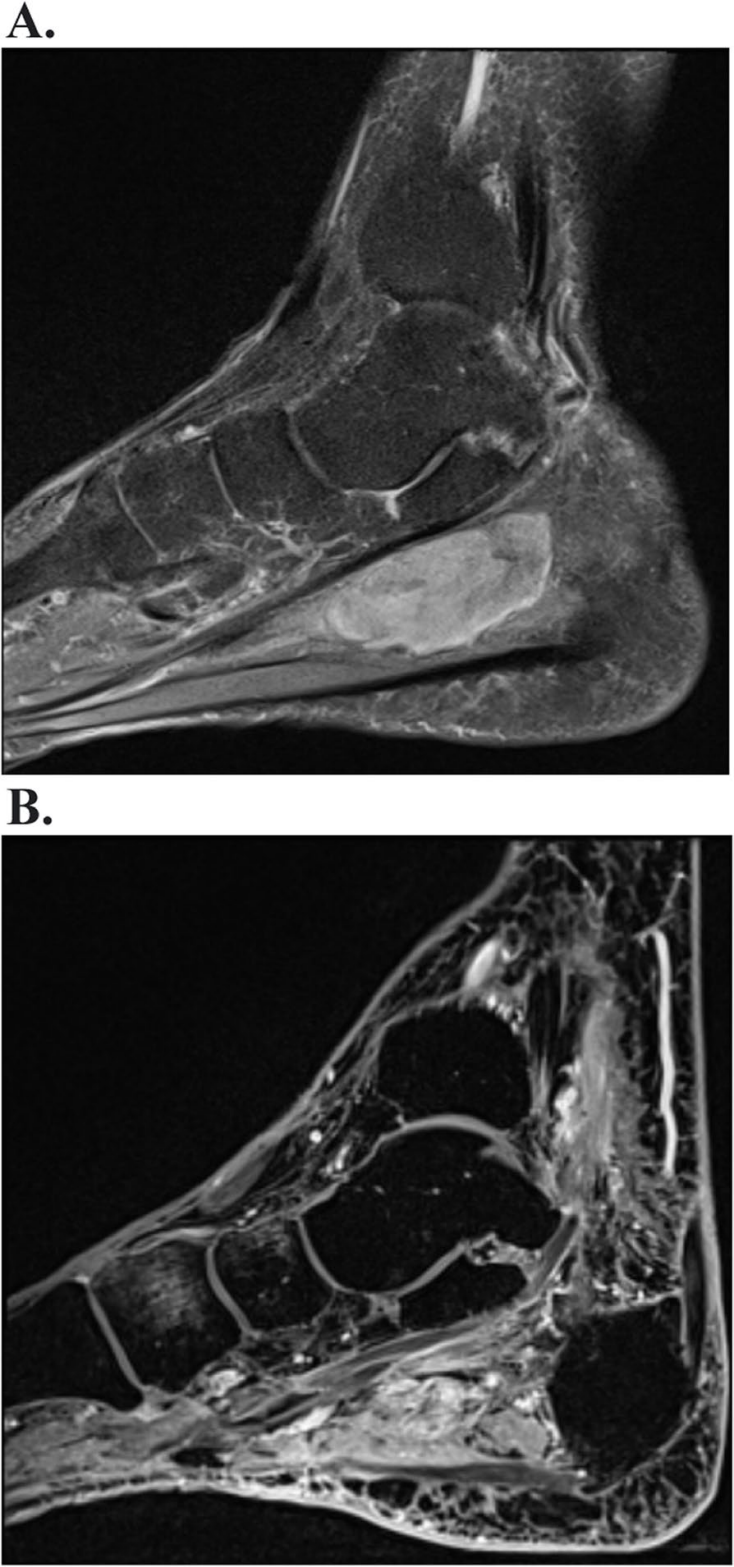
**A** Preoperative MR examination of the left ankle. Imaging in the sagittal plane (PD TSE sequence) with the finding of an oval-shaped soft tissue tumorous mass along the course of the tibial nerve. **B** Postoperative MR examination of the left ankle. Imaging in the sagittal plane (T1 VIBE sequence, after administration of contrast material) showing postoperative changes, without evidence of macroscopic residual tumor

After the initial neurological examination, the patient was sent for an ultrasound examination of the ankle, which showed a lobular mass measuring 50 × 22 × 16 mm. After supplementing the magnetic resonance imaging, the finding appeared to be a suspicious neurinoma of the tibial nerve (Fig. 1A).

**Fig. 2:**
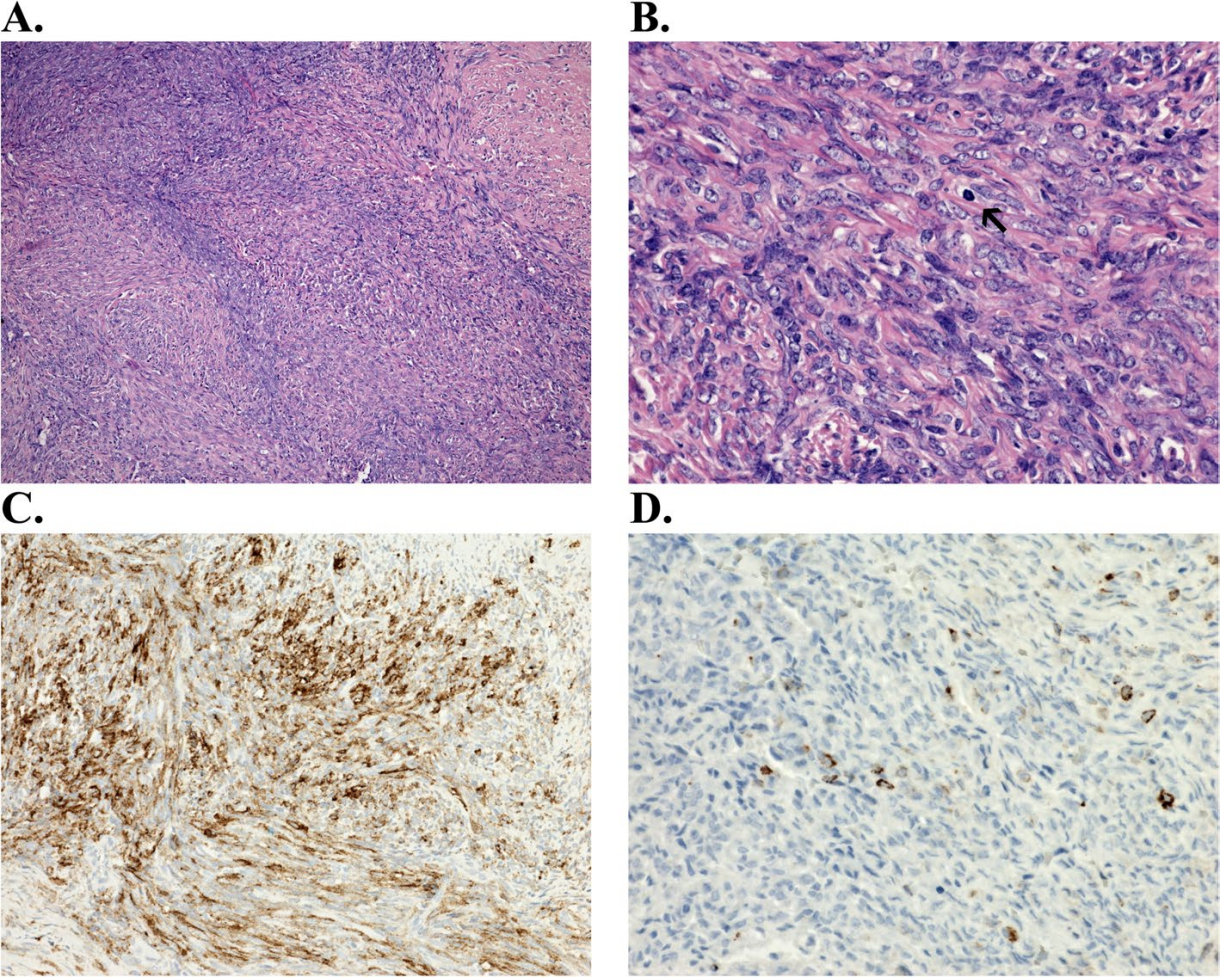
**A** Histological evidence of a spindle cell mesenchymal affection consisting of cells in poorly defined fascicles, with variably collagenized stroma. **B** Uniform appearance of the cells, with nuclei with fine chromatin, small nucleoli, without significant cytological atypia, with well-identifiable mitotic activity (mitosis indicated by an arrow). **C** Positivity in the epithelial membrane antigen test. **D** Single positivity in the detection of cytokeratin AE1/3

**Fig. 3:**
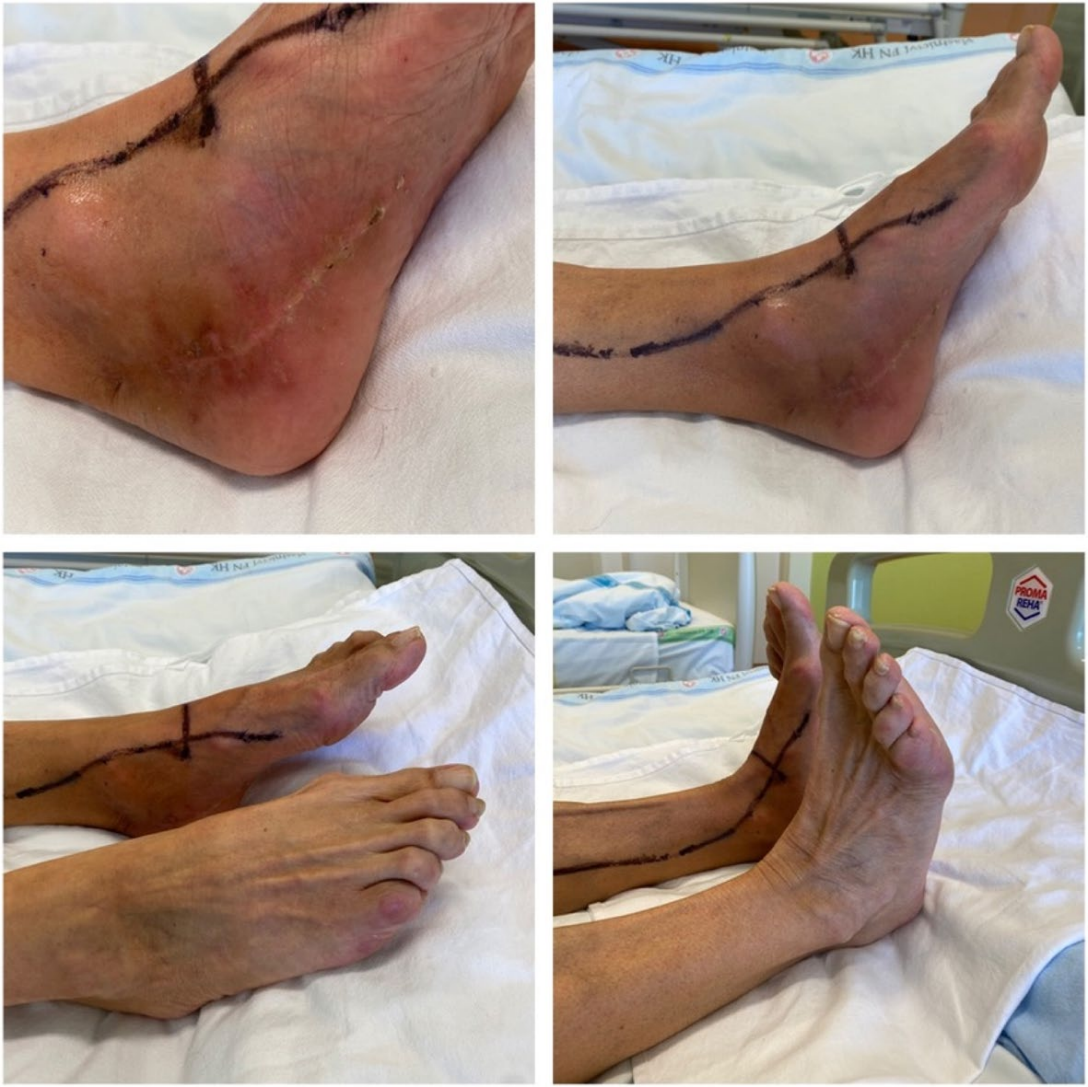
In the upper part, a healed surgical wound, in the lower part, a high-quality execution of plantar and dorsiflexion

The tumor was surgically excised, without prior biopsy verification: a 50 × 20 mm tumor was dissected in the distal part of the tarsal canal, which grew through the structure of the tibial nerve and in some places into the surrounding area and appeared intraoperatively as a neurofibroma. The nerve fascicles were freed from the tumor and the tumor was radically resected. Histologically, it was a spindle cell mesenchymal affection consisting of cells in poorly defined fascicles, with variably collagenized stroma (Fig. 2A). The cells had a uniform appearance, with nuclei with fine chromatin, small nucleoli, without significant cytological atypia, with well-identifiable mitotic activity (Fig. 2B, mitosis indicated by an arrow). Most cells were positive for epithelial membrane antigen (Fig. 2C) and isolated cells for cytokeratin AE1/3 (Fig. 2D) and S100 protein. Using a break-apart FISH probe, a break in the SS18 gene, a genetic alteration diagnostic for synovial sarcoma, in this case classified as monophasic synovial sarcoma, was demonstrated in the tumor. Tumor structures reached the resection margin; however, the tumor sample was fragmented and the assessment of the resection margin was not reliably possible for these reasons. Subsequently, a control MRI was performed, where postoperative changes, edema and scarring were displayed on the medial surface of the foot, no nodular solid tumor residue was found (Fig. 1B). For the histological findings and therefore the possible microscopic residue, the patient was indicated for a wide reexcision of the skin with the subcutaneous tissue of size 91 × 20 × 15 mm, which took place in January 2022. Histologically, the sarcoma structures were no longer found in the resection margins. CT of the body was without signs of disease dissemination. After the removal of the tumor, the patient was significantly relieved of pain. Two months after the second procedure, an electromyographic examination was completed, where a low summation muscle potential (CMAP) amplitude of the tibial nerve (1 mV) was measured on the left. Otherwise the findings were normal, thus indicating a partial axonal lesion of the tibial nerve. Now the patient is being treated with external radiotherapy to the tumor bed. The mobility of the leg lying on the bed is unimpaired and the tibial paresis is not convincingly expressed. The surgical wounds healed well (Fig. 3). The patient is now starting to try walking with her feet.

## Discussion and conclusions

Synovial sarcomas are malignant mesenchymal tumors, i.e., tumors arising from connective tissue. Benign mesenchymal tumors include, for example, fibroma, lipoma, chondroma, osteoma, myoma (leiomyoma or rhabdomyoma), angioma. Malignant mesenchymal tumors are called sarcomas.

Each peripheral nerve consists of axons that wrap around Schwann cells (here schwannomas, neurofibromas, or malignant tumors arise from the sheath of the peripheral nerve). The first protective covering, the endoneurium, is found around the Schwann cell, made of reticular and collagen fibers. Several bundles of such fibers are then enveloped by a second envelope, a peri-neurium formed from epithelioid cells, from which, for example, perineurinomas can arise. The last sheath is the epineurium, formed by a fibrous sheath of fibrous tissue, which wraps the bundles of fibers and fills them with mesenchymal cells, and from where sarcomas can originate [5]. Synovial sarcomas account for 5–10% of all soft tissue sarcomas [6]. However, the name synovial sarcoma is misleading, because the tumor does not originate from synovial cells, but rather from primitive mesenchymal cells [7]. The name most likely originated from the localization around the large joints on the limbs, more often on the lower ones, in the area of the knee joints [8].

There are generally two types of synovial sarcoma. Monophasic sarcoma, i.e., made up of only one type of cells, namely spindle or glandular cells, and biphasic sarcoma made up of both types of cells. Soft tissue sarcomas are usually fast-growing, painless tumors, but synovial sarcomas often grow more slowly, are painful, can cause joint contractures, swelling, and the average time from the onset of symptoms to the established diagnosis is 2 years [9]. Pain was the dominant symptom in our patient as well. It took approximately a year from the first symptoms to establish the diagnosis. Another distinguishing feature of synovial sarcomas from other sarcomas is the younger age at the time of the first symptoms - it often appears in adolescents and young adults, and affects both sexes equally [10, 11]. The basis for diagnosis is a magnetic resonance examination and then a biopsy, which should be performed before surgery so that a sufficiently radical procedure can then be performed. Cytogenetically, synovial sarcoma is characterized by the chromosomal translocation t(X;18)(p11.2;q11.2), which leads to the fusion of two genes - SS18 (SYT) on chromosome 18 and SSX on chromosome X, and to the formation of SYT-SSX fusion transcripts (SS18-::SSX1, SS18-::SSX2 and rarely SS18::-SSX4). Even in our patient, FISH analysis confirmed a break in the SS18 gene, testifying to this chromosomal translocation.

The immunohistochemical profile of synovial sarcomas characteristically shows signs of epithelial differentiation, even in the case of the monophasic form. The detection of pancytokeratins, positive in up to 90% of cases [12], and the expression of the epithelial membrane antigen (EMA) are typical. Both of these markers can be commonly identified in the vast majority of epithelial tumors and for example, also in rare epithelioid sarcomas [13]. Positivity in the context of monomorphic spindle cell sarcoma is, however, completely typical for synovial sarcoma. EMA positivity itself is non-specific in soft tissue pathology and can be encountered, for example, in perineuriomas or low-grade myxoid fibrosarcoma. The same is true of pancytokeratins, which can often be identified in, for example, angiosarcomas, chordomas, leiomyosarcomas and other soft tissue tumors [14]. For these reasons, it is necessary to assess the immunoprofile always in the context of the morphological findings. A relatively specific and sensitive immunohistochemical marker of synovial sarcoma is TLE1 [15], while immunohistochemical detection of other proteins (Bcl2, CD56 and CD99) is currently only of limited importance due to low specificity. Conversely, the absence of STAT6 immunoreactivity, typical for solitary fibrous tumors with pathognomonic NAB2::STAT6 fusion, is significant. Potentially problematic in the differential diagnosis is the focal positivity of the S100 protein, which can be identified in approximately 1/3 of synovial sarcomas [16]. S100 protein is a typical marker of neuroectodermal tumors and a similar focal positivity (in contrast to the diffuse positivity characteristic of malignant melanomas, neurofibromas and schwannomas) is often observed in malignant tumors from peripheral nerve sheaths, which also share a similar spindle cell morphology with synovial sarcomas. The most reliable means to distinguish between the two units is the proof of a break in the SS18 gene using FISH, RT-PCR or the massively parallel sequencing method [7].

The main treatment of synovial sarcoma is surgical treatment, which should take place only after a biopsy with histological examination of the tumor so that it is sufficiently radical and does not have to undergo an additional reoperation, as happened in the case of our patient.

Primary less radical excision without prior biopsy verification leads to a higher risk of local recurrence, even if a proper reexcision was performed immediately after biopsy verification of the sarcoma [17, 18].

Neoadjuvant radiotherapy is recommended especially for larger tumors (> 5 cm) or in cases where the resection margin has been reduced, for example due to an ongoing vessel, nerve or bone [19]. However, radiotherapy seems to prolong the overall survival of patients [20–22].

Synovial sarcomas appear to be more chemosensitive than other soft tissue sarcomas. In general, chemotherapy is recommended for younger patients, in whom it is expected to have a greater effect or for patients with unresectable or already generalized sarcomas [23–25].

Patient’s prognosis is uncertain. It is generally reported that the five-year survival is around 30–50% and only 10% of patients survive more than ten years. Negative prognostic factors include tumor invasion into bone or blood vessels, advanced age, larger tumor size, and less radical excision [26–28]. And as was written above, primary insufficiently radical excision, even with subsequent re-excision, increases the risk of local recurrence and thus worse progosis.

This case report draws attention to a rare type of malignant nerve tumor, which both clinically and radiologically can mimic benign peripheral nerve sheath tumors. Synovial sarcoma should be considered in very painful resistances, typically located around the joints of the lower limbs, the growth of which can be slow. Because the size of the tumor is a negative prognostic factor, it is necessary to make a timely diagnosis using MR imaging and a biopsy with histological examination and to start treatment quickly.

## Data Availability

Not applicable.

## Abbreviation

SS: Synovial sarcoma
